# Identifying the source populations supplying a vital economic marine species for the New Zealand aquaculture industry

**DOI:** 10.1038/s41598-023-36224-y

**Published:** 2023-06-08

**Authors:** Romain Chaput, Calvin N. Quigley, Simon B. Weppe, Andrew G. Jeffs, João M. A. C. de Souza, Jonathan P. A. Gardner

**Affiliations:** 1 School of Biological Sciences, Victoria University of Wellington, Wellington, New Zealand; 2 Cawthron Institute, Nelson, New Zealand; 3 MetOcean Solutions, Division of Meteorological Service of New Zealand, Raglan, New Zealand; 4 School of Biological Sciences, University of Auckland, Auckland, New Zealand

**Keywords:** Biooceanography, Conservation biology, Ecological modelling, Ocean sciences, Marine biology, Physical oceanography

## Abstract

Aquaculture of New Zealand’s endemic green-lipped mussel (*Perna canaliculus*) is an industry valued at NZ$ 336 M per annum and is ~ 80% reliant on the natural supply of wild mussel spat harvested at a single location—Te Oneroa-a-Tōhē—Ninety Mile Beach (NMB)—in northern New Zealand. Despite the economic and ecological importance of this spat supply, little is known about the population connectivity of green-lipped mussels in this region or the location of the source population(s). In this study, we used a biophysical model to simulate the two-stage dispersal process of *P. canaliculus*. A combination of backward and forward tracking experiments was used to identify primary settlement areas and putative source populations. The model was then used to estimate the local connectivity, revealing two geographic regions of connectivity in northern New Zealand, with limited larval exchange between them. Although secondary dispersal can double the dispersal distance, our simulations show that spat collected at NMB originate from neighbouring mussel beds, with large contributions from beds located at Ahipara (southern end of NMB). These results provide information that may be used to help monitor and protect these important source populations to ensure the ongoing success of the New Zealand mussel aquaculture industry.

## Introduction

Aquaculture of many marine species relies on seed supply from wild populations, either by harvesting juveniles directly from natural stocks or collecting settling larvae following natural spawning events^1,2^. This reliance means that the success of the aquaculture industry is tied to the resilience of natural populations and to the consistent (predictable) recruitment of juveniles following larval dispersal^3,4^. Understanding larval dispersal and the connections amongst populations is, therefore, key to the sustainable management of many aquacultures and fisheries resources^5–7^.

The green-lipped mussel *Perna canaliculus* (Gmelin 1791) is endemic to New Zealand and is the country’s most valuable aquaculture species, with an export value of NZ$ 336 M in 2019–2020^8^. Currently, the industry is largely reliant on the harvest of wild spat (post-metamorphic mussels usually < 10 mm shell length) that are collected and transported to aquaculture centres around the country where it is used to seed the farms^9^. The majority of the spat supply, about 80%, is harvested from beach-cast at one location: Te Oneroa-a-Tōhe—Ninety Mile Beach (henceforth NMB), in northern New Zealand^10^. The remainder is caught on spat catching ropes deployed in areas of natural high settlement or produced by small scale commercial hatcheries^11^. Despite their importance for the aquaculture industry, the source populations providing the spat at NMB, collectively known as Kaitaia spat, remain unknown^6,12^. Identifying the source populations will allow assessment and monitoring of their status, which may lead to their protection, and will help ensure the continuing success of the New Zealand mussel aquaculture industry^6,10^.

Green-lipped mussels are most commonly found in the northern and central parts of New Zealand, living in dense beds in the shallow sub-tidal environment and in aggregations in the intertidal environment^13–15^. *P. canaliculus* have a pelagic larval stage with a complex settlement behaviour which sees the settled spat detach and move repeatedly in search of a suitable habitat in which to establish^16^. Mussel larvae settle preferentially onto fragments of branching macroalgae, sediment, plant material and hydroids (i.e., a primary settlement phase), forming a complex mix of organic material including the mussel spat^9,17,18^. These macroalgae/spat mixtures, which are negatively buoyant due to the weight of the spat, tend to form aggregations (balls or rolls) that drift across the seafloor until they are either washed onto a beach, like NMB, or deposited on more suitable habitat for the mussels (e.g., established mussel beds or hard substrata). Mussel spat can then detach from the mixture to re-settle (i.e., a secondary settlement phase), a step which can happen multiple times and last up to a month before recruitment into adult mussel beds^9,11,16^. At NMB, this two-phase settlement process involving the macroalgae/spat is most pronounced and must be taken into account to identify the source populations of the spat supply.

The dispersal of mussel larvae and spat can be studied indirectly using Lagrangian based numerical models that aim to reproduce their trajectories from a source population to a settlement area^19,20^. Alternatively, when the source locations are unknown, particles may be backtracked from the settlement areas by following the current fields back in time. This approach has been used to study the effect of oceanographic structure on dispersal^21,22^, to reveal source populations^23,24^, or to reconstruct the pathways followed by larvae^25^. In the present study, we developed a realistic Lagrangian dispersal model^26^ to backtrack the mussel spat collected at NMB to their source populations, a method used successfully to identify source areas for green-lipped mussel spat in a different part of New Zealand^27^. Our model runs on a recently developed regional oceanographic model for New Zealand^28^ and accounts for the factors known to influence the transport of larvae and spat, such as the coastal circulation at NMB, local meteorological forcings, and the complex dispersal and settlement dynamics of green-lipped mussels^29–31^.

**Figure 1:**
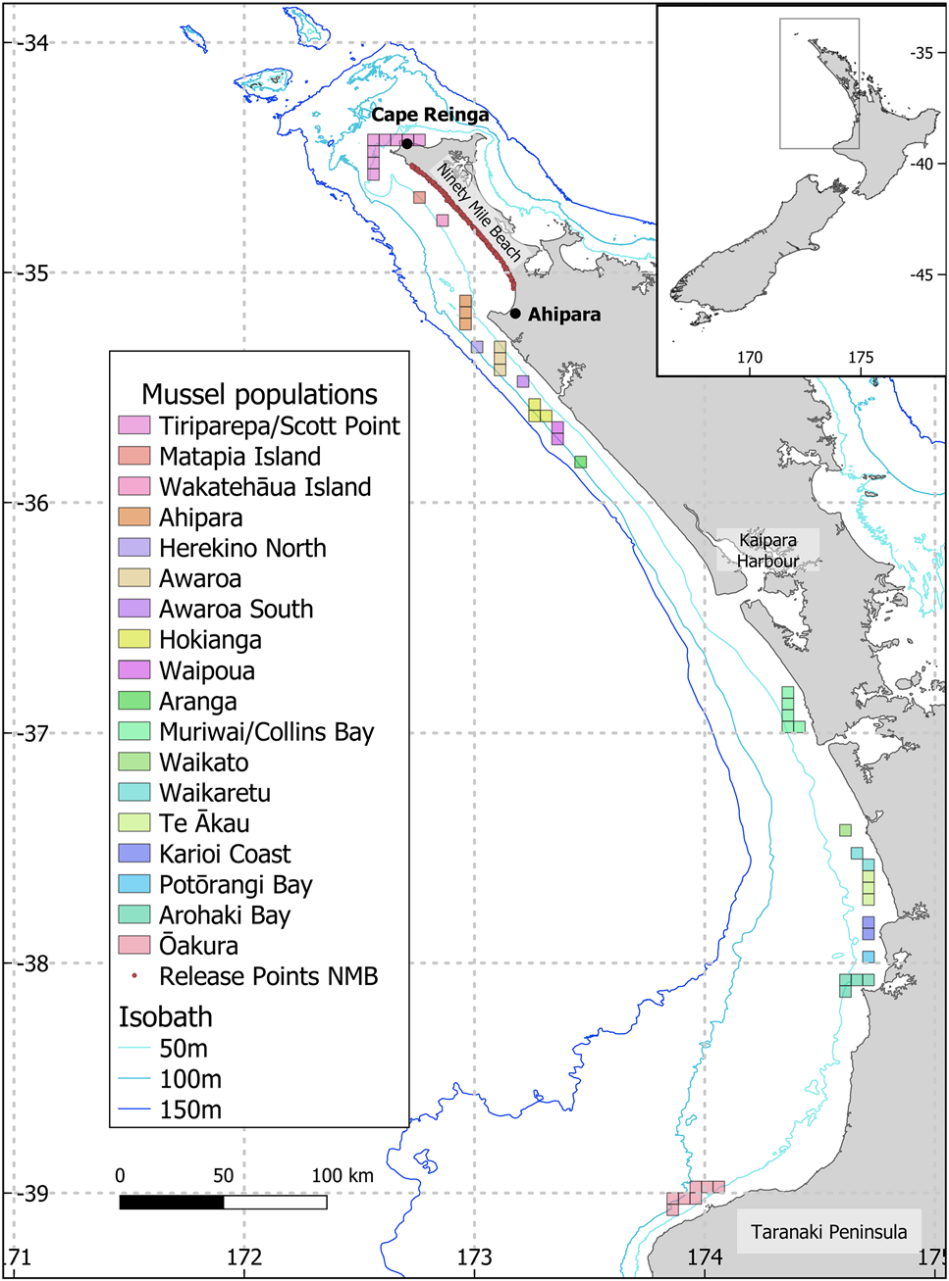
Potential mussel populations along the west coast of New Zealand North Island based on habitat suitability. The rocky shore was visually identified on satellite images and divided into 18 distinct geographic areas (from south to north): Ōakura, Arohaki Bay, Potōrangi Bay, Karioi Coast, Te Ākau, Waikaretu, Waikato, Muriwai/Collins Bay, Aranga, Waipoua, Hokianga, Awaroa South, Awaroa, Herekino North, Ahipara, Ninety Mile Beach South (Wakatehāua Island), Ninety Mile Beach North (Matapia Island), and Tiriparepa/Scott Point. Each population is subdivided in $5 \times 5$ km sites (49 sites in total).

Backtracking trajectories can be as simple as following streamlines backward in time^32^. In this case, all currents of the oceanographic model are reversed and the positions of the particles are advected backward over a time-step. However, this simple approach does not simulate turbulence unresolved by the oceanographic model which can increase the uncertainties of the estimates^24,33^, but including randomness to backtracking simulation requires specific considerations^32^. Therefore, to decrease the uncertainties we supplemented the backtracking estimates with a forward-tracking model integrating turbulence. This model was then used to investigate the regional population connectivity of *P. canaliculus* (Fig. 1) and compared to earlier genotypic analyses conducted on local mussel populations for validation^6.^

Several studies have shown a correlation between the dispersal estimates of biophysical models and genetic analyses because connectivity amongst populations is determined by the exchanges of individuals and therefore of genes^20,34,35^. Strongly connected populations are likely to be genetically similar (at least when using neutral genetic markers), while barriers to dispersal will lead to genetic divergence over time. Previous work attempted to identify the source populations of Kaitaia spat using genetic markers^36^, elemental signatures^37,38^, or a combination of both^6,12^. However, these studies either did not find any genetic structure amongst the different populations of the North Island^36^ or reported limited regional differences^6,12^. The present paper focusses on comparing regional connectivity modelled estimates with the results of genetic analyses to provide new insights into the mechanisms influencing the genetic structure of mussel in the NMB region and to help identify the source populations of the NMB spat supply. Altogether, our findings may be used to help safeguard the future of New Zealand’s green-lipped mussel aquaculture industry.

## Results

We carried out four different, but related, numerical experiments combining backward and forward particle tracking. We used this approach to take into account the different dispersal stages (larvae and spat) of *P. canaliculus*. Experiments were conducted to (1) identify the primary settlement areas of mussels harvested at NMB, (2) identify the source sites supplying spat to NMB, (3) estimate the regional connectivity amongst wild populations (Fig. 1), and (4) estimate the regional secondary connectivity.

### Experiment 1: identification of the primary (larval) settlement areas

**Figure 2:**
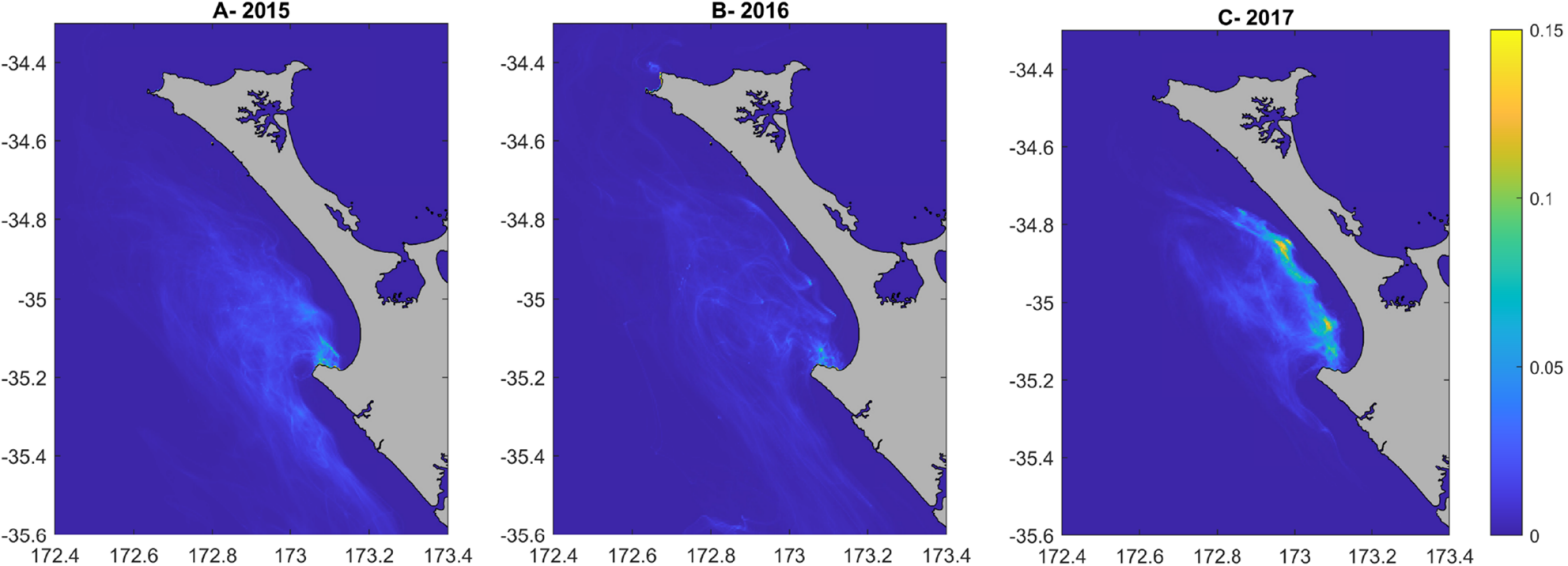
Probability density functions of backtracked trajectories. The yearly probability density of backtracked trajectories between 10 and 25.5 days of dispersal represented for the years (**A**) 2015, (**B**) 2016 and (**C**) 2017. Particles, representing macroalgae/spat, were released from 200 points spread randomly along NMB. Estimates from mussel spat collected at the beach put the dispersal time since primary settlement between 10 and 25.5 days. A lag of 0–5.5 days is applied depending on the release latitude of the particles since the further North the spat are collected on the beach the older the mussels are. Maps generated using Matlab version 9.13 (R2022b)^39^.

**Figure 3:**
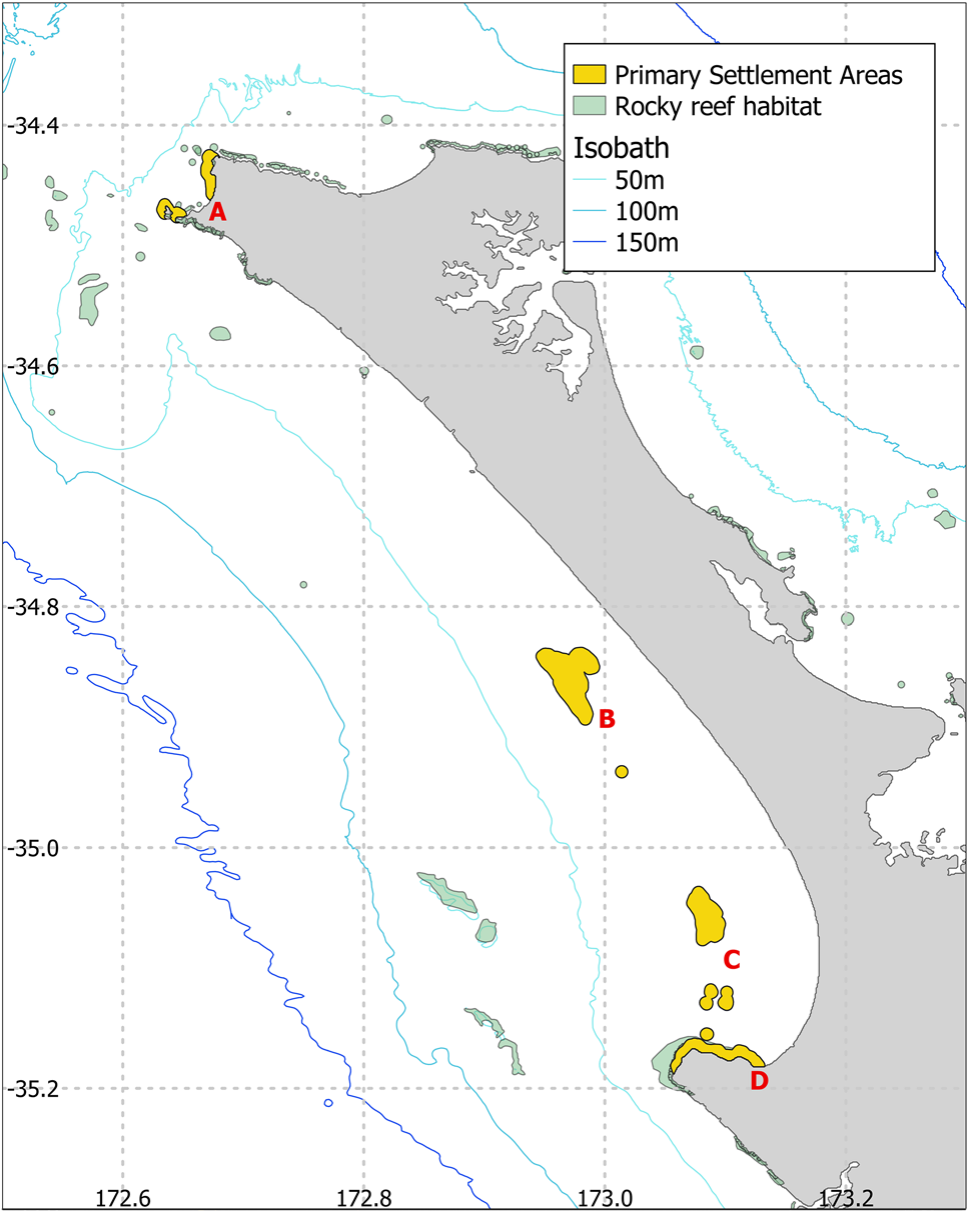
Potential locations of the primary settlement areas. The densities of backtracked trajectories of macroalgae/spat seeded on NMB in 2015–2017 were used to identify the primary settlement areas where mussel larvae could settle in large quantities onto macroalgae and other biological material. We identified four possible locations (labelled in red on the map): (**A**) Tiriparepa/Scott Point, (**B**) offshore of NMB, (**C**) Ahipara Bay, and (**D**) Te Kōhanga/Shipwreck Bay.

Maps of the yearly probability density functions of the trajectories of the macroalgae/spat, aggregated across the 5 months of release, for the three years of runs (2015, 2016, 2017) showed that trajectories aggregated in the southern region of NMB (Fig. 2). There were important inter-annual differences in the position and extent of these high probability areas. In 2015, the only area where trajectories aggregated was within Te Kōhanga/Shipwreck Bay with the densest area covering 9946 km$^{2}$ (Fig. 2A). In 2016, there was an aggregation near the coast at Ahipara, covering 9565 km$^{2}$, and an aggregation near Tiriparepa/Scott Point, in the northern part of the region, covering 7157 km$^{2}$ (Fig. 2B). In 2017, there were five areas of aggregations: one close to the shore at Ahipara (12,311 km$^{2}$), one within Te Kōhanga/Shipwreck Bay (3759 km$^{2}$), and three further north and offshore of NMB (totaling 23,327 km$^{2}$) (Fig. 2C). Extracting the densest parts of trajectory aggregations for the three years highlighted four locations that could be candidates for the primary settlement areas (labelled on Fig. 3): Tiriparepa/Scott Point (7157 km$^{2}$-A), alongshore NMB (23,327 km$^{2}$-B), Ahipara Bay (12,311 km$^{2}$-C), and nearshore at Te Kōhanga/Shipwreck Bay (15,397 km$^{2}$-D).

### Experiment 2: identification of the source mussel populations

**Figure 4:**
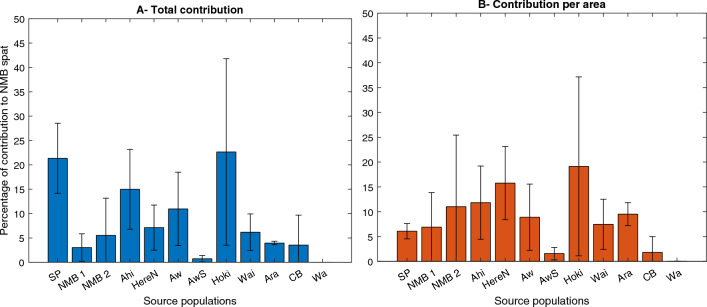
Source populations of the NMB spat. Average percentage of contributions (**A**) per population and (**B**) normalised per geographical area (in square kilometres), to the NMB spat supply for the years 2015–2017. Error bars represent the standard deviation for each population. The source populations are all in the Northern part of the study domain (north to south): Tiriparepa/Scott Point (SP), Matapia Island (NMB1), Wakatehāua Island (NMB2), Ahipara (Ahi), Herekino (HereN), Awaroa (Aw), Awaroa South (AwS), Hokianga (Hoki), Waipoua (Wai), Aranga (Ara), Muriwai/Collins Bay (CB), and Waikato (Wa).

Analysis of the second backtracking experiment showed that only 11 of 18 populations, all located in the northern part of the domain, contributed spat to NMB: Tiriparepa/Scott Point, Matapia Island, Wakatehāua Island, Ahipara, Herekino, Awaroa, Awaroa South, Hokianga, Waipoua, Aranga, Muriwai/Collins Bay, and Waikato (Fig. 4A). The southernmost, and farthest from NMB, source populations connected by larval and spat dispersal were Muriwai/Collins Bay, with 3.5% of averaged contribution, and Waikato, with 0.006% of averaged contribution. Both contributions were only observed in 2016. For 2015 and 2017, the southernmost population reached was Aranga (overall contribution of 4%).

A one-way ANOVA revealed that there was a statistically significant difference amongst the source populations in contribution to the NMB spat (F(10, 22) = 2.71, *p* = 0.0246). Hokianga, Tiriparepa/Scott Point, Ahipara, and Awaroa were the main source sites with a combined averaged contribution of 69.9% across all years. The largest inter-annual variability was found for Hokianga (refer to error bars on Fig. 4) due to a contribution of 44.7% in 2015, but only 13.2% in 2016, and 10% in 2017. Tiriparepa/Scott Point, Ahipara and Awaroa together contributed to almost half (47%) of the spat supply and provided a more stable supply from year to year.

Because the spawning biomass of mussels at each site is unknown, the site-specific contributions to the NMB spat supply were also normalised by site geographical area in the model (in square kilometres on Fig. 4B). The main contributors by area to the NMB spat supply were Hokianga, Herekino North, Ahipara, and Wakatehāua Island (NMB 2). There was large inter-annual variability for both Hokianga (contribution mostly in 2015) and Wakatehāua Island (contributions of 27.3% in 2017, 5.7% in 2016, 0% in 2015). The next largest contributions were from Herekino North and Ahipara. The one-way ANOVA revealed that there was no statistically significant difference in normalised contribution amongst the source populations (F(10, 22) = 1.2, *p* = 0.3447).

### Experiment 3: regional connectivity

**Figure 5:**
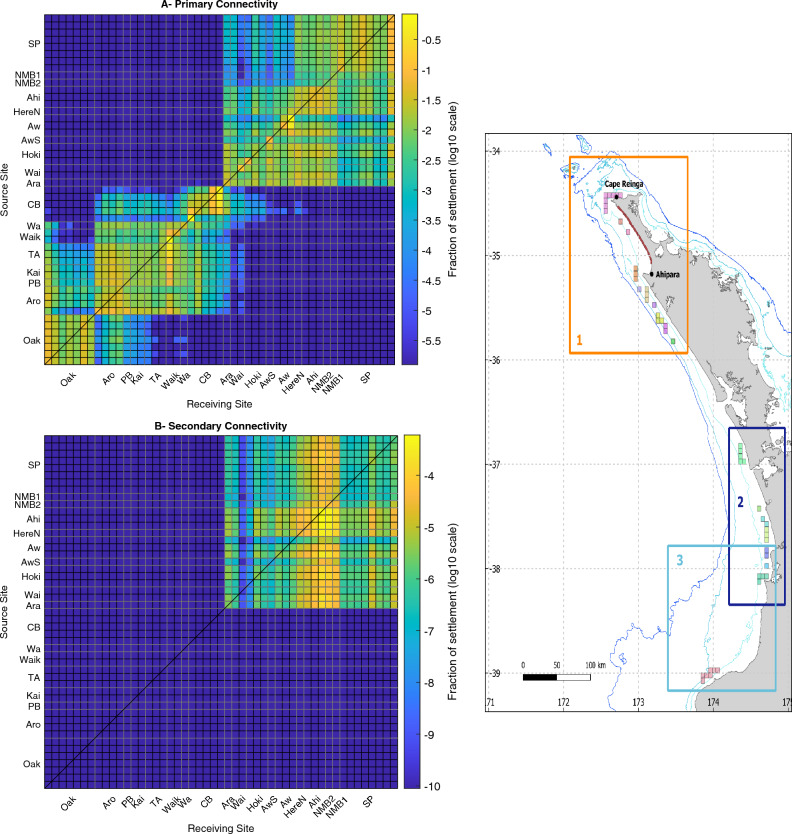
Decadal regional connectivity of *P. canaliculus*. (**A**) Primary and (**B**) secondary connectivity matrices for the western coast of the North Island of New Zealand presenting the 10-year averaged proportion of larvae/spat dispersing from sources sites (y-axis) to receiving sites (x-axis). Right panel locates the northern connectivity block (1), and southern connectivity block (2–3) on the domain map. Sites are grouped in 18 mussel populations (from south to north): Ōakura (Oak), Arohaki Bay (Aro), Potōrangi Bay (PB), Karioi Coast (Kai), Te Ākau (TA), Waikaretu (Waik), Waikato (Wa), Muriwai/Collins Bay (CB), Aranga (Ara), Waipoua (Wai), Hokianga (Hoki), Awaroa South (AwS), Awaroa (Aw), Herekino North (HereN), Ahipara (Ahi), Wakatehāua Island (NMB2), Matapia Island (NMB1), and Tiriparepa/Scott Point (SP). Local retention is represented on the diagonal, while southward and northward connectivity are represented above and below the diagonal, respectively. The fraction of settlement is presented as a logarithmic scale.

**Figure 6:**
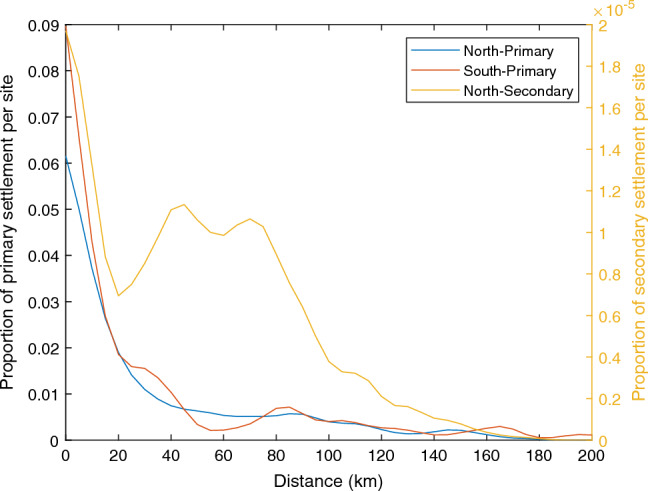
Regional dispersal kernels. Dispersal kernels showing the proportion of settlement at a given distance from the release site. Kernels are shown for the northern and southern blocks of primary connectivity (blue and red lines, respectively), and for the secondary connectivity (yellow line). Note that kernels for primary and secondary dispersal are shown using different scales on the y-axis.

Analysis of the decadal connectivity matrix (Fig. 5A) revealed a pattern of spatially-explicit connectivity composed of two blocks with limited exchange between them. The northern block extended from Tiriparepa/Scott Point in the north to Aranga in the south and contained NMB (Fig. 5, label 1 on right panel). In this block, all sites were interconnected with bidirectional exchanges, that is, a similar degree of northward and southward connectivity. The mussel populations with the highest degree of local retention were Awaroa and Ahipara. In this block, the highest source and receiving population was Tiriparepa/Scott Point ($1.9\times 10^{5}$ and $2.2\times 10^{5}$ larvae per year, respectively), followed by Awaroa ($1.5\times 10^{5}$ and $1.5\times 10^{5}$ larvae per year, respectively). The dispersal kernel for the northern block of connectivity showed a single peak of settlement within the first 20 km from the release sites (Fig. 6).

A second block of connectivity was located in the southern part of the domain and extended from Muriwai/Collins Bay to Ōakura (Fig. 5, labels 2–3 on right panel). Connectivity within this block was heterogeneous. First, there was a well connected group of sites located from Muriwai/Collins Bay to Arohaki Bay, with slightly more northward than southward connectivity (Fig. 5, label 2 on right panel). Second, the population of Ōakura had more southward connectivity, and more distant connections, than northward connectivity (Fig. 5, label 3 on right panel). In this second block of connectivity, there was important local retention in the populations of Muriwai/Collins Bay and Waikato. In this block, the highest source and receiving population was Muriwai/Collins Bay ($2.8\times 10^{5}$ and $3.0\times 10^{5}$ larvae per year, respectively), followed by Te Ākau as source ($1.2\times 10^{5}$ larvae per year) and Waikaretu as sink ($1.9\times 10^{5}$ larvae per year). The dispersal kernel for this block of connectivity reflected this heterogeneity with a primary peak of settlement within the first 20 kilometers and a secondary peak of settlement around 80 km (Fig. 6).

Exchanges between the two major blocks of connectivity were limited and unidirectional. Most exchanges happened via Muriwai/Collins Bay which contributed a small number of larvae to Aranga, Waipoua, Hokianga, and Awaroa, and very few larvae further north to Herekino North/Ahipara. Some populations in the second connectivity block, from Waikato to Arohaki Bay, also contributed a few larvae to the nearest populations in the first connectivity block (Aranga and Waipoua). There were no connections going from the northern block to the southern block of connectivity.

### Experiment 4: secondary (spat) connectivity

The 10-year averaged secondary connectivity (Fig. 5B) was restricted to the northern block (Fig. 5, label 1 on right panel). Ahipara was the most important population, being both the largest source and a large sink of spat (321 and 174 per year respectively). In addition, Ahipara experienced strong local retention. Matapia Island and Wakatehāua Island contributed spat mostly to Ahipara, but otherwise acted as sink populations, receiving large numbers of settlers (319 and 216 per year respectively) from all the other populations. The secondary connectivity, however, did not reveal new connections amongst populations and, therefore, did not modify the overall pattern of regional connectivity created by the primary dispersal (Fig. 5A). The fraction of secondary settlement was also one order of magnitude less than for the primary connectivity. The dispersal kernel for the secondary dispersal shows a strong peak of settlement within the first 20 km and a secondary peak of settlement extending between 40 and 80 km, showing that the secondary dispersal can double the dispersal distance compared to the primary dispersal (Fig. 6).

### Comparing modelled connectivity to genetic connectivity

**Figure 7:**
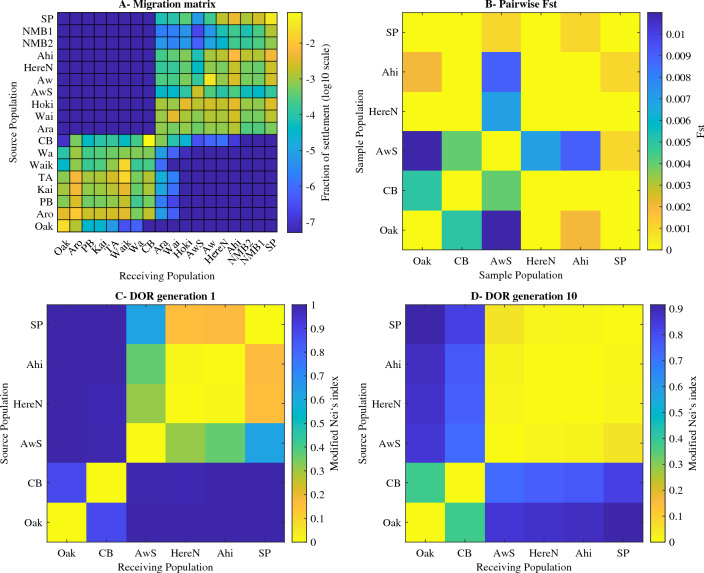
Modelled and genetic connectivities. (**A**) modelled migration matrix and (**B**) pairwise genetic connectivity estimates between the six sample sites. The migration matrix was used to compute the derived oceanographic resistance (DOR) for (**C**) one generation and (**D**) 10 generations. The DOR presents the modified Nei’s pairwise distances which can be directly compared to the pairwise Fst values (mirrored in this figure for ease of visualisation). The genetic analysis was conducted on microsatellite variation at 10 polymorphic loci^6^.

Genotypic analyses^6^ did not identify pronounced genetic structure amongst the six populations, with all pairwise estimates of $F_{ST}$ being $\le$ 0.011 (Fig. 7B). In contrast, the migration matrix (Fig. 7A) and DOR for one generation (Fig. 7C) showed evidence of structure, with high levels of similarity amongst the populations of Herekino North, Ahipara and Tiriparepa/Scott Point, and a lower level of similarity with Awaroa South, while the populations of Ōakura and Muriwai/Collins Bay appeared to be isolated. The DOR after 10 generations showed high levels of similarity between the populations in the northern block of connectivity and high levels of similarity between Ōakura and Muriwai/Collins Bay (Fig. 7D). The Mantel tests performed between the DOR matrices and the pairwise $F_{ST}$ matrix were both not statistically significant (correlation of $-\,0.0283$ with a *p*-value of 0.4963 for the DOR generation 1 and correlation of $-\,0.1470$ with a *p*-value of 0.6305 for the DOR generation 10). There was no evidence of a relationship between modelled and genetic connectivity.

## Discussion

This study is the first to attempt to identify the source populations of the Kaitaia spat with a biophysical model of dispersal. This numerical approach is increasingly being used to study the connectivity of marine populations^40^ and backtracking simulations have been proven useful to identify the origin of biotic and abiotic material^41–44^. However, these models must realistically represent the larvae biological and behavioural characteristics^45^. The biophysical model developed for this study is unique in that it represents the complex dispersal processes of both the larvae and macroalgae/spat mixture for *P. canaliculus*, and considers separately primary and secondary settlement. Simulating this two-step dispersal allowed us to map the regional connectivity amongst local populations and identify potential areas of primary settlement.

### Primary settlement areas

The backtracking simulations of the macroalgae/spat collected at NMB identified a few areas where the trajectories aggregated and where larvae could have settled. Some of the primary settlement areas identified in our study are located close to the coast, which supports the idea that mussel larvae recruit to attached macro-algae that then fragment due to the additional weight of the seed and drift toward NMB^30^. The rocky reefs located in Te Kōhanga/Shipwreck Bay and around Tiriparepa/Scott Point have been documented as receiving both mussel larvae and spat in large quantities during the spawning season^46,47^. Additional primary settlement areas were identified in the middle of Ahipara Bay and offshore of NMB, at the southern end of the beach. These areas do not correspond to known rocky reefs and could therefore indicate areas where mussel larvae and bottom-drifting macroalgae are accumulated by mesoscale eddies, a mechanism known to favour larval settlement^48^. This result is supported by observations of great concentrations of mussel larvae and loose lying macroalgae within Ahipara Bay^46^. This suggests that larvae could also settle offshore on drifting biological fragments coming from distant reefs.

The rocky reef areas identified in Te Kōhanga/Shipwreck and Ahipara Bay are common to all the years simulated and, therefore, are more likely to represent the actual primary settlement areas than the areas at Tiriparepa/Scott Point or offshore NMB, which were only observed in 2016 and 2017, respectively. However, even within this restricted area, the exact location of the primary settlement area is likely to vary from year to year depending on the patterns of winds, known to drive the variability of the NMB spatfall^30^. Strong winds and storms increase the production of drifting macroalgae whilst wind-driven currents favour the transport of macroalgae/spat along the shore and across the surfzone^49^. Understanding the dynamics behind the aggregation and transport mechanisms of macroalgae/spat will lead to better forecasting of the variability of spat supply at NMB.

### Estimated source populations

All of the mussel populations connected to NMB through the two-step dispersal process are located in the northern part of our domain and in the neighbourhood of the beach itself. The populations most likely to contribute to the NMB spatfall are located at Hokianga, Tiriparepa/Scott Point and Ahipara. There are large mussel beds in these three locations^13,50,51^. However, the mussel bed at Tiriparepa/Scott Point goes through pronounced density fluctuations, with mass mortality events attributed to the accumulation of macroalgae/spat rafts that out-compete the adult mussels^47^. Therefore, this population might not be a stable and reliable source of larvae for the NMB spatfall each and every year. Similarly, we estimate that Hokianga is an important contributor in certain years (e.g., 2015) but due to its large inter-annual variability, it might also not be a long term stable source of larvae. The spat supply contribution of Ahipara, when scaled up to reflect the size of the local mussel population (large intertidal and shallow subtidal beds), may be significant, and overall more stable in the long term. Furthermore, both backtracking and forward tracking simulations highlighted the contribution of Ahipara to the overall connectivity as an important source of larvae and macroalgae/spat. In conclusion, we suggest that the mussel population at Ahipara is likely to be the main contributor to the NMB spatfall events.

Our ability to correctly identify the source populations hinges on the accuracy of the oceanographic model, the uncertainties associated with particle tracking methods, and the appropriate representation of biophysical interaction in the model. While the oceanographic model’s accuracy has been tested for open waters and at meso-to-large scale, near-shore processes that are not well represented in the model may impact the results. A higher resolution model would better represent along and cross-shore transport of particles in the nearshore region, processes that are critical to spat transport, and could impact the retention of particles within Ahipara Bay^52,53^. Uncertainties in backtracking simulations could be reduced using complex approaches that better integrate stochastic movements which may lead to better estimates of the contributions of mussel populations to the NMB spat supply^32,54^. Finally, these estimates would benefit from a better knowledge of the exact location and extent of the mussel beds. Indeed, the model could over- or under-estimate the connectivity by considering mussel reefs in areas where they are absent or by not including large subtidal mussel beds if they were to exist in the region. Conducting targeted benthic surveys would help decrease this uncertainty.

### Regional connectivity

We identified two well-defined blocks of connectivity with limited exchange between them. The northern block extends from Tiriparepa/Scott Point (the northernmost population in our study) to Aranga and includes all of NMB. All the populations in this block are interconnected and we identified an area of strong local larval retention around Ahipara Bay. The southern block extends from Muriwai/Collins Bay to Ōakura. The exchanges within this block of connectivity are not as homogeneous as in the northern block, with potentially two sub-groups of connectivity: a northern, well-interconnected group of populations centered around Te Ākau, and a group around Ōakura characterised by a directionality of exchanges with stronger southward than northward connectivity. Dispersal kernels revealed a strong local retention with most settlement happening within 20 km of the release location. These results are in agreement with other studies that suggest that most mussel larvae likely disperse over short distances (10 km or less)^27,29,55^, and are also supported by analysis of the population genetic structure of *P. canaliculus* in New Zealand which reveals high rates of self-recruitment within populations, despite limited genetic structure^56^. Interestingly, while secondary dispersal can double the dispersal distances, it did not create new connections in the region.

The patterns of connectivity observed here can be explained by the influence of the currents on larval dispersal (Supplementary Fig. 1). Specifically, the northern block of connectivity is associated with strong meso-scale eddies around Ahipara Bay that may retain and recirculate larvae in the area. The southern block of connectivity seems to be shaped by the meanders of the Westland Current that help retain larvae at the coast. We suggest that the separation between the two main blocks of connectivity is explained by an absence of suitable mussel habitat. Habitat discontinuity is a well-known driver of population structure and/or differentiation^57,58^. Ripiro Beach, the longest sandy shore in New Zealand at 107 km in length, is located between Muriwai and Aranga, and there is no intertidal or subtidal rocky habitat that could support green-lipped mussels in this region. In addition, an alongshore north-flowing current prevents larvae from the northern populations travelling to the southern areas. It does, however, allow some larvae to travel from Muriwai/Collins Bay to the northern populations.

### Genetic studies and future modelling perspectives

The model suggests the presence of two major subpopulations of green-lipped mussels on the west coast of the northern North Island, with limited exchange between them. However, this result is in contrast to findings from studies of the population genetics of *P. canaliculus* at the national or regional scale^6,56^, where the only genetic differentiation reported appears just south of Cook Strait, separating the North Island and South Island, similar to the genetic structure found in many coastal species^59^. The absence of genetic structure amongst northern populations suggests high levels of connectivity with little differences amongst populations at the regional scale. This disparity could be due to sufficient gene flow between the two subpopulations and to the uncertainties in the model outputs. Typically, panmixia can be maintained despite barriers to dispersal given a small number of migrants per generation and stepping stone populations^60–62^. The application of new genetic markers, such as Single Nucleotide Polymorphisms (SNPs), with higher resolution ability^63,64^ than mitochondrial DNA SSCPs^36^ or microsatellite loci^6^, are expected to be better suited to test the model of two regional groups because they are more adapted to study the structure of populations of highly connected species with large effective population sizes^65^.

Additionally, our study only considered the primary spawning season producing the spat harvested at NMB between July and November. However, green-lipped mussels can spawn and settle throughout the year^66^ and, although the magnitude of the reproductive output is lower than during the primary spawning season, they contribute to population connectivity. Seasonal primary and secondary settlement patterns could be substantially different and could account for the mismatch between model estimates and genetic structure. However, modelling the trickle spawning of *P. canaliculus* showed very little variability in seasonal dispersal patterns at the national scale^20^. Overall, linking genetic structure to modelled estimates is complicated because dispersal and settlement of larvae may be decoupled^67,68^. Ecological factors not considered in the model, such as food availability, predation rates, and post-settlement survival, may impact genetic connectivity independently of oceanographic factors, creating a mismatch between larval dispersal and realised connectivity^69^. Overall, further research needs to be conducted to better understand whether patterns of spatial genetic structuring are caused by oceanographic processes^5,59,70^.

A multi-disciplinary approach combining genetic analysis and biophysical modelling is key to addressing some of the challenges faced by sustainable exploitation of marine resources in the world^2,71^. If applied to other regions of New Zealand, the method employed in our study can be used to inform local spat-catching operations and decrease the reliance of the industry on the NMB spatfall events^27^. It can help in finding areas where spat are most likely to accumulate and therefore may be collected on settlement ropes, and it can help in identifying local source populations that may need protection and/or restoration. Our study shows, however, that high spatial resolution models are necessary to fully understand the dispersal and connectivity of populations at a local scale. Oceanographic models with still greater resolution may be required to explain the observed intra-annual and inter-annual variability of spat supply, a result that may lead to better predictions of spatfall events at NMB^30,72^. A better understanding of the mechanisms involved in spat settlement, retention, and arrival at NMB may also explain and help predict the infrequent periods when spat have historically not arrived at the beach, which would be invaluable to the New Zealand green-lipped mussel industry by allowing better planning of spat collection activities^73^.

## Materials and methods

### Study area

Ninety Mile Beach (NMB) is located on the western coast of the north of Te-Ika-a-Maui, New Zealand’s North Island (Fig. 1). The wave-exposed sandy shore stretches mostly in a north-to-south direction over 88 km, from Ahipara (southern end) to Tiriparepa/Scott Point (northern end). While the oceanography over the north-east shelf of New Zealand has been well described, little is known about the north-west shelf and its currents^74,75^. The continental shelf is narrower at the northernmost point (Cape Reinga/Te Rerenga Wairua) with a width of 10 km, and wider at the southern end in the Taranaki region with a width of 100 km^75^.

The major currents in the northern region are influenced by the Tasman Front, a series of eddies and meanders originating from the East Australian Current (EAC). At Cape Reinga the EAC bifurcates and flows southward to form the East and West Auckland Currents either side of the Northland peninsula^76^. The West Auckland Current (WAC) is weaker and more variable than its eastern counterpart^74,75^. The WAC flows north to south from Tiriparepa/Scott Point to Raglan where it meets the north-flowing Westland Current^77^. A full description of the currents is available in the supplementary material (Supplementary Fig. 2). The region of NMB is dominated by wind-driven currents and strong tides^74^. Inshore currents (<200 m water depth) flow north, as evidenced by the movement of mussel spat that appear first in Ahipara and drift north following NMB^46^. The mean wind stress at NMB is cross-shore, which could create this alongshore flow and a region of upwelling^74^.

The domain of interest extends over 600 km from Ōakura in the south to Tiriparepa/Scott Point in the north (Fig. 1). This domain matches the region used in a recent study of green-lipped mussel spatially-explicit genetic and shell microchemistry variation^6^. There is no published database of the location of wild mussel beds in New Zealand. However, we know that *P. canaliculus* is abundant on North Island coasts^13^, so we considered that every rocky shore is a potential habitat for green-lipped mussels. The rocky shore along the coast was visually identified on satellite images and divided into distinct geographic areas of continuous stretches of rocky shore. We identified 18 potential mussel populations (Fig. 1) that were subdivided into 49 sites of $5 \times 5$ km (in accordance with the oceanographic model resolution). These sites were used in the model both as spawning points and settlement areas for the mussel larvae.

### Hydrodynamic model

The oceanic conditions were provided by the Moana Hindcast Model^28^, an hydrodynamic developed by MetOcean Solutions using the Regional Ocean Modeling System (ROMS) version 3.9^78^. The Moana Hindcast is a large regional model that covers New Zealand coastal and shelf circulation at a grid size of $5 \times 5$ km with 50 vertical layers ($\sigma$-coordinate system), and an hourly temporal resolution. The model grid extends from 52° S to 31° S and from 161° E to 185° E. The model implementation and evaluation against available observations are described in detail in^28^. The current study uses data between June and November (the main spawning period of *P. canaliculus*) for a 10-year period (2008–2017). The hydrodynamic model takes into account atmospheric forcing (10m winds, humidity, air temperature and sea level pressure), provided by the Climate Forecast System Reanalysis (CFSR), National Center for Atmospheric Research (NCAR). Other forcings include fluxes from 42 rivers around New Zealand (climatological data from data.govt.nz portal) and tides (harmonics provided by the TPXO global tidal solution^79^).

### Lagrangian particle tracking model

We used OpenDrift (http://github.com/opendrift), an open-source Python-based framework for Lagrangian particle modelling^26^, to simulate the dispersal trajectories of mussel larvae and spat around NMB. We used an OpenDrift sub-module developed specifically for bivalve larvae^20^ and configured it to represent the dispersal stages of *P. canaliculus*.

The model was used to track the dispersal of particles (i.e., larvae and spat) over the austral winter and spring (June to November) to match the spat harvesting season at NMB^10,18^. Larval settlement takes place 3 to 5 weeks after spawning^66,80^ and depends on environmental conditions, settlement cues, and settlement substratum availability^16,81,82^. Therefore, we tracked larvae for a maximum of 35 days, while the competency for settlement started 21 days after release and was restricted to the potential habitat identified on Fig. 1. Outside the competency period and/or the availability of settlement habitat, larvae that intersected with the coastline or bottom were moved back to their previous position. Besides active settlement, it is unknown whether *P. canaliculus* larvae exhibit active swimming behaviours during their dispersal, though New Zealand bivalve larvae seem to be able to control their vertical distribution^83^ and a previous modelling study found good agreement between observations and dispersal estimates when considering a simple vertical sinking velocity^84^. We modelled the dispersal of larvae using a vertical sinking velocity of 0.001 $\hbox {m}\hbox {s}^{-1}$, a value within the range reported for bivalve larvae^85^.

OpenDrift was run offline, and the motion of the particles was integrated using a 4th order Runge–Kutta method with a time-step of 900 s. We performed a set of four experiments: two backtracking experiments (to identify the origin of the spat collected on NMB) and two forward tracking experiments (to estimate the regional connectivity). In both cases, the motion of the particles was integrated in three dimensions. For the backtracking experiments, we did not integrate diffusion (nor vertical sinking velocity) to the model. For the forward tracking experiments, unresolved turbulence was modelled using a horizontal diffusion coefficient of 0.1176 $\hbox {m}^{2}\hbox {s}^{-1}$ and a vertical diffusion coefficient of 0.01 $\hbox {m}^{2}\hbox {s}^{-1}$, the latter computed over a 90 s time-step (based on parameters previously used to model bivalve larvae dispersal^84,86^).

In total, we released ~ 60 million particles between the months of June and November over the years 2008–2017. The number of particles released was scaled up from the minimum necessary to saturate the system^20^. Release sites -mussel reefs, primary settlement areas, and NMB itself- were selected to represent mussel occurrence (Fig. 1) and dispersal potential. As detailed below we carried out four different, but related, experiments to test for connectivity and to identify source sites. Input parameters for each experiment are detailed in Table 1 and the experiment configurations are described below.

**Table 1:** Modelling parameters for the experiments.

	**PLD**	**Competency**	**Release** **depth**	**Horizontal and ** ** vertical diffusion**	**Release ** ** area**	**Settlement ** ** area**	**Release ** ** points**	**Months**	**Years**	**Experiment ** ** type**
**Experiment 1**	20 days (+ lag)	10 days (+ lag)	seafloor + 1 m	No	Ninety Mile Beach	–	200	July–November	2015–2017	Backtracking
**Experiment 2**	35 days	21 days	0–30 m	No	Primary settlement areas	Source populations	200	July-November	2015–2017	Backtracking
**Experiment 3**	35 days	21 days	0–20 m	Yes + vertical sinking: 0.001 m/s	Regional populations	Regional populations + Primary settlement areas	980	June-October	2008–2017	Forward tracking
**Experiment 4**	30 days	10 days	seafloor + 1 m	Yes + vertical sinking: 0.001 m/s	Primary settlement areas	Regional populations	200	July–November	2008–2017	Forward tracking

Input parameters for the 4 experiments, i.e., 2 backtracking to identify the source populations and 2 forward tracking to identify the regional connectivity. In each experiment, 1000 particles were released from each release point for each month. The release depth, release locations and settlement locations changed depending on the life stage modelled in the experiment.

### Experiment configurations

#### Experiment 1: identification of the primary (larval) settlement areas

The trajectories of the spat were backtracked from NMB to potential areas where mussel larvae undergo their primary settlement. We released 200,000 particles per month from 200 points randomly distributed along NMB (from 173.18° E, 35.07° S to 172.73° E, 34.53° S, Fig. 1). Particles were released linearly in time from the months of July to November, inclusive, and years 2015 to 2017, inclusive (in backward time). Particles were released 1 m above the seafloor to reproduce the bottom-drifting dispersal of spat^46^. Mussel seed collected at NMB are estimated to be between 10 and 20 days old. However, mussels collected in the southern part of the beach (near Ahipara) are smaller and younger than mussels collected in northern part of the beach^46^. Using the size of mussel spat sampled from different locations along NMB^46^ and the corresponding spat growth rate^87^, we estimated that the dispersal time (spat age) difference from one end of the beach to the other is ~ 5.5 days. Therefore, we applied a dispersal lag to the spat depending on their release latitude (from 0 to 5.5 days from south to north) and backtracked the spat for a maximum of 25.5 days. Primary settlement areas were located using the density of trajectories between 10 and 25.5 days of dispersal (see following section for the details of the analysis).

#### Experiment 2: identification of the source mussel populations

Mussel larvae were backtracked from the primary settlement areas, identified in experiment 1, to their source populations. We released 200,000 particles per month from 200 points randomly distributed within the primary settlement areas. Particles were released linearly in time from July to November, inclusive, and years 2015 to 2017, inclusive (in backward time). Particles were released randomly from a depth of 0 to 30 m (vertical distribution range of newly settled spat in an ongoing experiment in Bay of Plenty, pers. com. Wenjie Wei), and backtracked for a maximum of 35 days. Dispersal trajectories were stopped when larvae that were aged from 21 to 35 days moved on top of one of the 18 potential mussel populations (Fig. 1). Because the experiment was backtracking larvae, the final positions correspond to the (putative) source populations. After 35 days of dispersal, all larvae still remaining in the water column were removed. The contributions to the spatfall were compared using an ANOVA performed on the sites connected to NMB.

#### Experiment 3: regional connectivity

To estimate the regional connectivity, we tracked the dispersal of mussel larvae amongst mussel populations, including the primary settlement areas as possible settlement grounds. For this experiment, we released 20,000 larvae per month from 20 points distributed randomly within each site (total of 980,000 larvae per month between the 49 sites, see Fig. 1). Larvae were released linearly in time from June to October, inclusive, for the years 2008 to 2017, inclusive. Larvae were randomly released between the depth of 0 to 20 m (common distribution range of mussel beds), and tracked for a maximum of 35 days. Larvae were given a vertical sinking velocity of 0.001 $\hbox {m}\hbox {s}^{-1}$ during dispersal. Larvae were allowed to settle during their competency period (21 to 35 days after release) if on top of one of the 49 sites or one of the primary settlement areas. Larvae that did not settle after the 35 days PLD were removed from the simulation.

#### Experiment 4: secondary (spat) connectivity

To estimate the secondary connectivity, we tracked the dispersal of mussel spat from the primary settlement areas to the mussel populations. We released 200,000 particles per month, linearly in time, between July and November, inclusive, from 200 points randomly distributed within the primary settlement areas identified in the first experiment. Particles were released 1 m above the seafloor. Particles were allowed to settle after 10 days of dispersal (in accordance with the minimum age of mussel seeds collected on NMB) and were tracked for a maximum of 30 days. Spat that did not settle after 30 days were removed from the simulation. The particles were given a terminal sinking velocity of 0.001 $\hbox {m}\hbox {s}^{-1}$ to reproduce the observed negative buoyancy of the drifting material containing the spat.

### Connectivity estimates analysis

#### Metrics of biophysical connectivity

Dispersal—the sum of larval hatching, transport, survival, and settlement^88^, to which we add the transport of spat—was analysed using three metrics: the probability density functions of trajectories, connectivity matrices and dispersal kernels.

The probability density functions of trajectories were estimated by computing the probability of presence of the particles within the cells of a 299 $\times$ 299 matrix (~ 0.14 $\hbox {km}^{2}$ per cell) covering our domain of interest. This analysis was conducted on the first backtracking experiment to find the primary settlement areas. Only the portion of trajectories with particles aged 10 to 20–25.5 days after release were considered for the analysis. To find where trajectories aggregated, we extracted the areas with the highest densities of particles using an arbitrary threshold density of 0.6 particles km$^{-2}$. A buffer of 500 m was then added to the high density areas to account for modelling uncertainties and widen the areas to seed particles.

The connectivity matrix^89^ represents the probability of connection between mussel populations using a matrix ${\mathcal {C}}$ of size $n \times n$ (where $n=49$, the number of sites) with source sites on the y-axis and sink sites on the x-axis. Each element ${\mathcal {C}}_{ij}$ indicates the fraction of settlement (number of settlers divided by total release) between a source *j* and a settlement-site *i*, with local retention indicated on the diagonal ($j=i$). A logarithmic scale was used to highlight differences amongst populations. The third experiment produced a regional connectivity matrix for each of the 10 years simulated. The matrices were then averaged to estimate the decadal regional connectivity matrix. Variability in connectivity was studied using Empirical Orthogonal Functions (EOF) that decomposed the inter-annual variance in modes and related it to the populations on the connectivity matrix (Supplementary Fig. 1).

Secondary connectivity was presented using the product of two rectangular matrices: a 49 $\times$ 5 connectivity matrix with the 49 source sites on the y-axis and the 5 primary settlement areas on the x-axis, resulting from the third experiment, and a 5 $\times$ 49 connectivity matrix with the primary settlement areas on the y-axis and the potential sink sites on the x-axis, resulting from the fourth experiment. The product of these two matrices showed the site-to-site connectivity via the primary settlement areas. The addition of the primary and secondary connectivity matrices resulted in the estimate of total connectivity for *P. canaliculus*.

The dispersal kernel of a given site measures the probability that a larva born at that site settles at *x* km from it. A dispersal kernel can be analysed in a variety of ways depending on the focus of the study^90^. Here, we segregated the dispersal kernels based on the analysis of the primary and secondary connectivity matrices.

#### Comparing modelled connectivity to genetic connectivity

A genotypic analysis was conducted as part of a previous study aiming to identify the source of the NMB spat (detailed methods and results are presented in^6^). Population genetic structure, based on microsatellite variation at 10 polymorphic loci, was assessed for 288 mussels sampled at six sites (Tiriparepa/Scott Point, Ahipara, Tanutanu, Mitimiti, Whatipū, and Ōakura, corresponding respectively to Tiriparepa/Scott Point, Ahipara, Herekino North, Awaroa South, Muriwai/Collins Bay, and Ōakura in the present study), and used to compute pairwise $F_{ST}$ values.

We tested for a correlation between genetic empirical data and connectivity estimates following^91^. The modelled estimates of connectivity were transformed into a metric of distance that could be compared to the population differentiation ($F_{ST}$ values from^6^). The averaged connectivity matrix was down-scaled to the population level—with 19 populations *p*– and standardised across columns to generate a migration matrix. This matrix quantifies the relative contributions of all sources to the total settlement at all receiving populations, and its diagonal represents self-recruitment. The migration matrix was then projected forward in time^92^ to simulate exchanges amongst mussel populations over 10 generations.

Both migration matrices were then used to compute Nei’s $D_{A}$ genetic distance^93,94^ such that $D_{A} = 1-\sum ^{p}_{k=1}{\sqrt{X_{ik}\times Y_{ik}}}$, where *x* and *y* are migration probabilities between pairs of populations. The resulting distance matrices, named Derived Oceanographic Resistance (DOR), are square diagonal matrices with pairwise distances normalised between 0 and 1 (with 0 on the diagonal). The correlation of genetic distances ($F_{ST}$) between pairs of the six sites with the corresponding DOR was tested with Mantel tests for similarity matrices based on 5,000 permutations^20^.

## Supplementary Information

Supplementary Information.

## Data Availability

The data underlying this article will be shared on reasonable request to the corresponding author. The results of the Moana Hindcast model are open-access and available for a period of 28 years (1994–2020) at https://www.moanaproject.org/hindcast. Opendrift, the open-source Lagrangian model used for the simulations, is made available at http://github.com/opendrift. The bivalve module used specifically to simulate the dispersal of green-lipped mussel larvae and spat is open-source and available at http://github.com/metocean/opendrift/blob/master/opendrift/models/bivalvelarvae.py.
