# Supplementary material for: Identifying the source populations supplying a vital economic marine species for the New Zealand aquaculture industry
Source: Sci Rep. 2023 Jun 8;13:9344. doi: 10.1038/s41598-023-36224-y (PMC10250383; doi:10.1038/s41598-023-36224-y)
Supplement: Supplementary file 1 — Supplementary Information. [file 41598_2023_36224_MOESM1_ESM.pdf]

Supporting Information for
Identifying the source populations supplying a vital economic marine species for the New Zealand aquaculture industry

Romain Chaput[1,2,*], Calvin N. Quigley[1], Simon B. Weppe[3], Andrew G. Jeffs[4], Joao M.A.C. Souza[3], Jonathan P.A. Gardner[1]

May 21, 2023

1. School of Biological Sciences, Victoria University of Wellington, Wellington, New Zealand
2. Cawthron Institute, Nelson, New Zealand
3. MetOcean Solutions, Division of Meteorological Service of New Zealand, Raglan, New Zealand
4. School of Biological Sciences, University of Auckland, Auckland, New Zealand
∗ Corresponding author; e-mail: romain.chaput@cawthron.org.nz

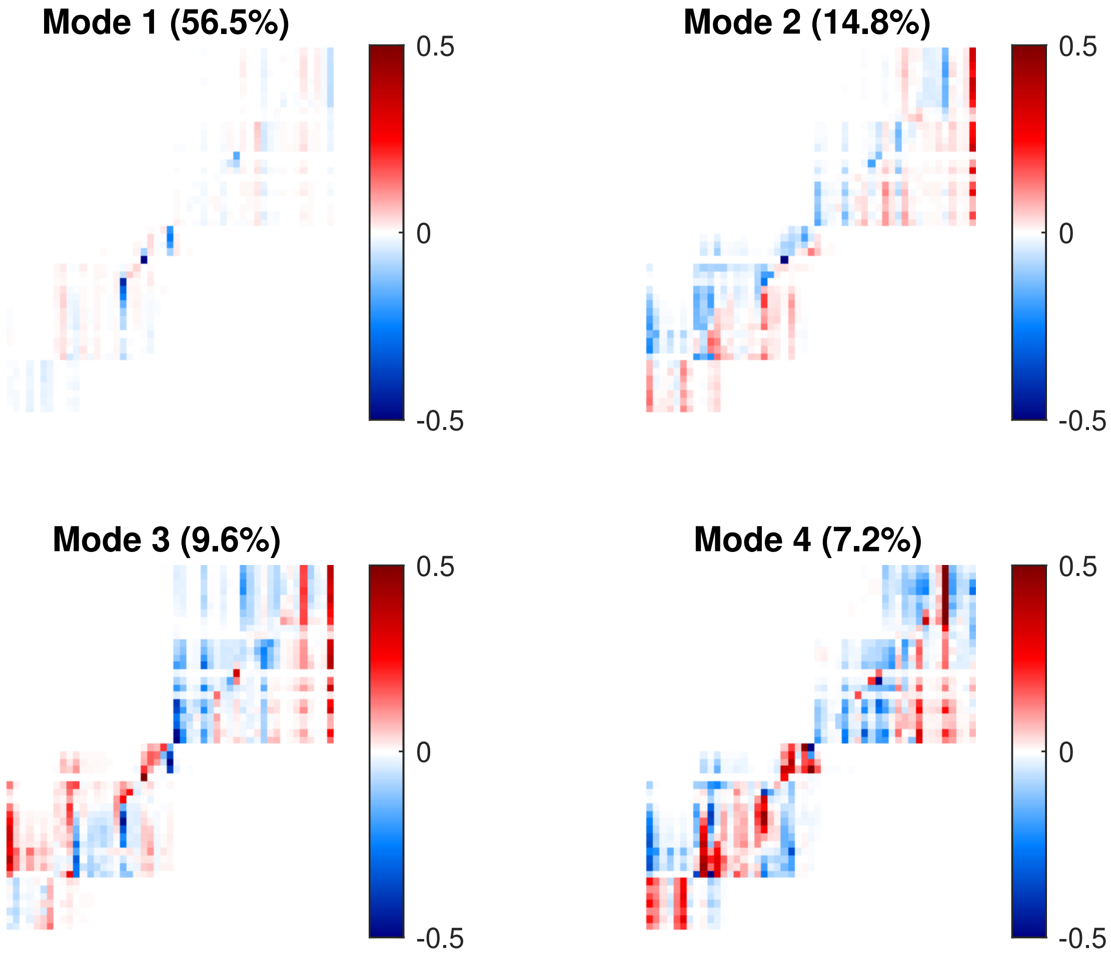

Figure 1: **EOF modes for the regional connectivity.** Spatial and temporal patterns of settlement variability in the regional connectivity over the 10 years covered by the simulations. The first EOF accounted for 56.5% of the variance and identified Muriwai/Collins Bay and Waikaretu as the populations with the highest variance of settlement, which was linked to local retention. The second mode of the EOF, which explained 14.8% of variance, highlighted the annual co-variability in southward/northward connectivity. The third mode of connectivity, accounting for 9.6% of variance, showed an alternation between southern and northern connectivity blocks. The remaining modes of the EOFs explained less than 19% of the settlement variability and represented stochastic variations in settlement.

Figure 2 shows the average surface currents during the months considered in this study. Larvae released in the northern half of the domain are advected northward by a coastal current flowing in this direction during the spawning season, following a possible extension of the Westland Current[1,2]. This current interacts with the Tauroa Peninsula and is pushed offshore, creating meanders and eddies along NMB and in Ahipara Bay itself. Larvae advected in the vicinity of NMB are thus retained by these strong mesoscale eddies and recirculated in the area, creating the northern block of connectivity. We did not observe a consistent presence of the southward-flowing West Auckland Current[1,3,2]. During our period of interest (from early winter to spring), the offshore currents appear to be unstructured and variable.The southern block of connectivity is shaped by the Westland Current that meanders close to the shore in the southern half of the domain, creating mesoscale eddies at the coast that retain larvae. The intensity and location of the current changes from month to month, but on average it transports larvae offshore toward the north while a weak inshore counter-current helps recirculate larvae from north to south between the Whakaki estuary and Raglan. Further south, inshore currents off Ōakura are influenced by the D'Urville Current[4], flowing toward the Cook Strait and transporting larvae south. The influence of the D'Urville Current could explain why Ōakura is slightly disconnected from the southern block of connectivity because mussel larvae from local reefs are more likely to be advected south into the Cook Strait rather than north into the Tasman Sea.

Currents off the west coast of the North Island are weak and dominated by variability with inshore flows driven by wind forcing[2]. The transport of mussel larvae and macroalgae/spat in this area is also strongly influenced by the wind[5,6]. Along NMB, the winds are predominantly onshore[7,2], creating an Ekman transport going north (to the left of the wind in the Southern hemisphere) parallel to the beach. This Ekman transport explains the observed northward transport of macroalgae/spat from Ahipara to Scott Point parallel to NMB. In addition, tides have a strong impact on the currents over the continental shelf, which is particularly wide in the area of NMB[8]. Nearshore currents are influenced by the tidal cycle, often reversing with the tides. These mechanisms are important for the transport of mussel larvae and macroalgae/spat, promoting populations connectivity within each of the two blocks of connectivity. Interestingly, despite this variability and the lack of well-defined structures in the currents, the simulated mussel larvae remain close to the coast during all the period of their dispersal, and this true over all of the domain of interest, with the exception of Scott Point where some larvae tend to be advected far offshore. Local currents seem to favour the retention of larvae in the nearshore environment, which is likely to be one of the factors responsible for the large quantities of mussel spat (more than 170 tonnes of macroalgae/spat per year[9]) that arrive consistently at NMB.

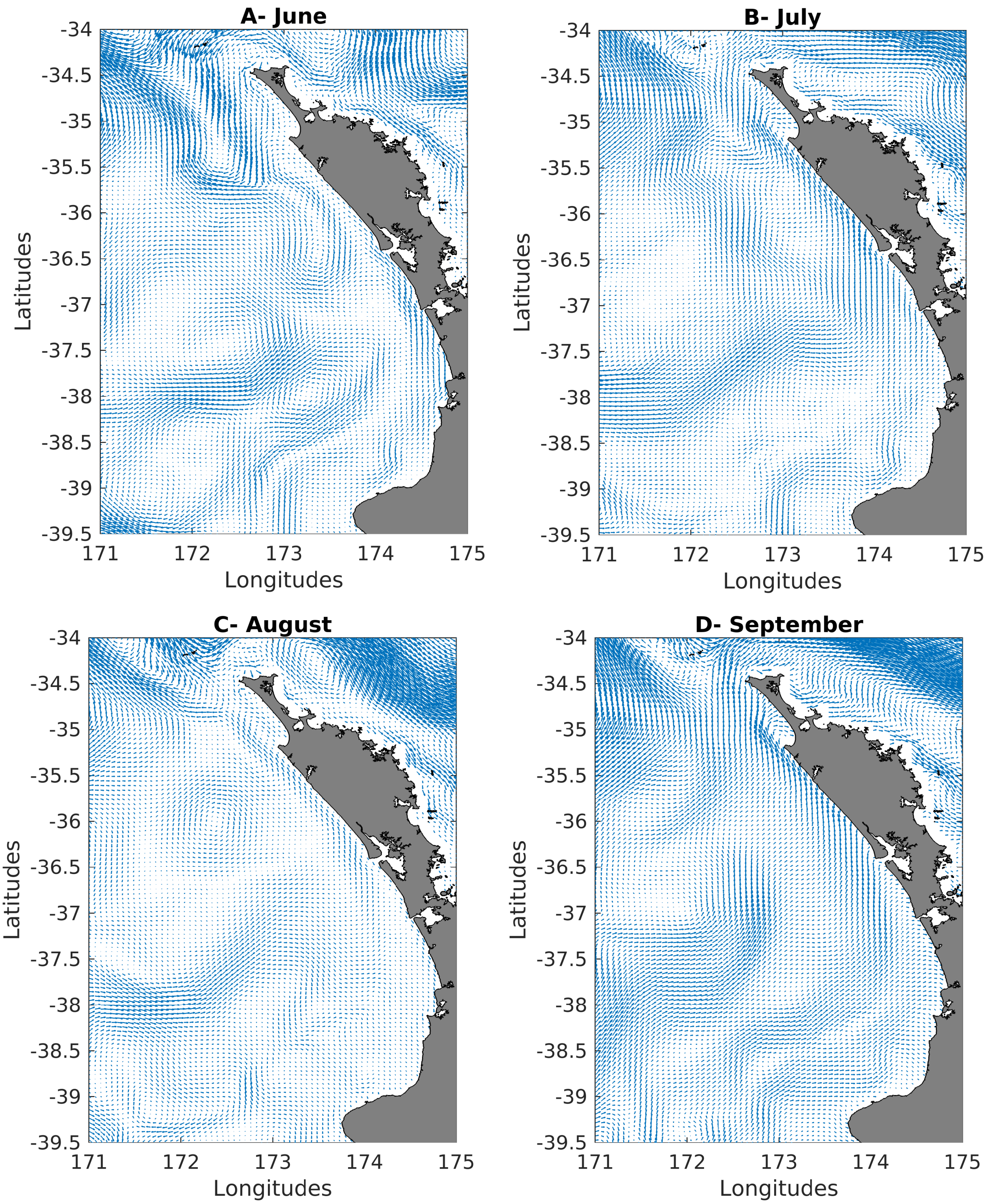

Figure 2: **Monthly averaged surface currents between 2008 and 2017.** Representation of the surface current vectors for u and v for the months of A-June, B-July, C-August and D-September averaged over 10 years (2008-2017) in the study area. Maps generated using Matlab version 9.13 (R2022b)[10].

References

[1] Brodie, J. W. Coastal surface currents around New Zealand. *New Zealand Journal of Geology and Geophysics* **3**, 235–252 (1960).

[2] Sutton, P. J. & Bowen, M. M. Currents off the west coast of Northland, New Zealand. *New Zealand Journal of Marine and Freshwater Research* **45**, 609–624 (2011).

[3] Ridgway, K. R. & Dunn, J. R. Mesoscale structure of the mean East Australian Current System and its relationship with topography. *Progress in Oceanography* **56**, 189–222 (2003).

[4] Heath, R. A. A review of the physical oceanography of the seas around New Zealand — 1982. *New Zealand Journal of Marine and Freshwater Research* **19**, 79–124 (1985).

[5] McQuaid, C. D. & Phillips, T. E. Limited wind-driven dispersal of intertidal mussel larvae: In situ evidence from the plankton and the spread of the invasive species Mytilus galloprovincialis in South Africa. *Marine Ecology Progress Series* **201**, 211–220 (2000).

[6] Demmer, J. *et al.* The role of wind in controlling the connectivity of blue mussels (Mytilus edulis L.) populations. *Movement Ecology* **10**, 1–15 (2022). URL `https://doi.org/10.1186/s40462-022-00301-0`.

[7] Alfaro, A. C., McArdle, B. & Jeffs, A. G. Temporal patterns of arrival of beachcast green-lipped mussel (*Perna canaliculus*) spat harvested for aquaculture in New Zealand and its relationship with hydrodynamic and meteorological conditions. *Aquaculture* **302**, 208–218 (2010). URL `http://dx.doi.org/10.1016/j.aquaculture.2010.02.028`.

[8] Chiswell, S. M., Bostock, H. C., Sutton, P. J. & Williams, M. J. Physical oceanography of the deep seas around New Zealand: A review. *New Zealand Journal of Marine and Freshwater Research* **49**, 286–317 (2015).

[9] Alfaro, A. C., Copp, B. R., Appleton, D. R., Kelly, S. & Jeffs, A. G. Chemical cues promote settlement in larvae of the green-lipped mussel, *Perna canaliculus*. *Aquaculture International* **14**, 405–412 (2006).

[10] MATLAB. *version 9.13.0 (R2022b)* (The MathWorks Inc., Natick, Massachusetts, 2022).
